# Innate immunity and monocyte-macrophage activation in atherosclerosis

**DOI:** 10.1186/1476-9255-8-9

**Published:** 2011-04-28

**Authors:** Joseph Shalhoub, Mika A Falck-Hansen, Alun H Davies, Claudia Monaco

**Affiliations:** 1 Cytokine Biology of Atherosclerosis, Kennedy Institute of Rheumatology, Faculty of Medicine, Imperial College London, UK; 2 Academic Section of Vascular Surgery, Department of Surgery and Cancer, Faculty of Medicine, Imperial College London, UK

**Keywords:** Atherosclerosis, Inflammation, Innate immunity, Toll-like receptors, Monocyte subsets, Macrophage subtypes, Macrophage polarisation

## Abstract

Innate inflammation is a hallmark of both experimental and human atherosclerosis. The predominant innate immune cell in the atherosclerotic plaque is the monocyte-macrophage. The behaviour of this cell type within the plaque is heterogeneous and depends on the recruitment of diverse monocyte subsets. Furthermore, the plaque microenvironment offers polarisation and activation signals which impact on phenotype. Microenvironmental signals are sensed through pattern recognition receptors, including toll-like and NOD-like receptors - the latter of which are components of the inflammasome - thus dictating macrophage behaviour and outcome in atherosclerosis. Recently cholesterol crystals and modified lipoproteins have been recognised as able to directly engage these pattern recognition receptors. The convergent role of such pathways in terms of macrophage activation is discussed in this review.

## Introduction

Atherothrombotic vascular disease is quickly becoming the leading cause of mortality worldwide, accounting for a fifth of all deaths [1]. The manifestations of the disease are often sudden and dramatic, including myocardial infarction and sudden death. Cerebrovascular atherothrombosis is responsible for ischaemic stroke, a major source of disability and dependence, and represents a rising health-economic burden [2].

Progress has been made in refining our understanding of the process of inflammation which underlies atherosclerosis since the early descriptions by Rudolf Virchow during the 19^th ^century [3,4] and subsequently Russell Ross in the late 1990s [5-8]. The development of an atherosclerotic plaque begins with the recruitment of blood-borne inflammatory cells at sites of lipid deposition [9] or arterial injury [5]. Local rheological factors, such as low and oscillatory (with vortices) blood-to-wall shear stress dictate the location of atherosclerotic plaques to characteristic points along the vasculature [10,11].

Atherosclerosis shares features with diseases caused by chronic inflammation [7]. Inflammation is intrinsically linked with disease activity, as the numbers of monocyte-macrophages infiltrating the plaque [12] and their location at plaque rupture-sensitive sites (such as the fibrous cap and areas of erosion [13,14]) is related to plaque vulnerability. Moreover, lymphocyte abundance and their activation markers relate to plaque activity [13]. Macrophage differentiation is acknowledged as critical for the development of atherosclerosis [15]. The intimate relationship between atherosclerosis and inflammation is further exemplified by the involvement of cytokines and chemokines at all stages of the process of atherosclerosis (reviewed in detail by [16]). The extent of the inflammatory infiltrates and their strategic location within the protective fibrous cap is associated with plaque rupture and/or thrombosis [17]. Adventitial inflammation has also been described [18], and is linked with an expansion of the adventitial *vasa vasorum *in unstable atherosclerosis [19]. The inflammatory nature of atherosclerosis is supported by the association between circulating plasma inflammatory markers, particularly C-reactive protein, with cardiovascular outcomes, even in the absence of dyslipidaemia [6]. Further evidence for a link between systemic inflammation and cardiovascular disease is the increased incidence of cardiovascular events in chronic inflammatory conditions, such as inflammatory arthritis and systemic lupus erythematosus [7,8]. The expanding knowledge base regarding inflammation in atherosclerosis has resulted in a keen interest in targeted therapeutics and functional imaging tools for the high-risk atherosclerotic plaque [20].

### Innate immunity is a key player in atherosclerosis

How is inflammation established and maintained within an atherosclerotic plaque? Inflammation in physiological conditions is a self-limiting ancient protective mechanism that defends the host from invading pathogens. It relies on two arms: innate immunity and adaptive immunity. Innate immunity is activated immediately upon encounter with the pathogen and is executed primarily by myeloid cells with the participation of some "innate" lymphocyte sub-populations. Adaptive immunity is a second line of defence that is based upon the generation of antigen-specific recognition apparatus at cellular (T cell receptor) and humoral (antibody) levels.

In the past decade it has become apparent that the innate arm of the immune inflammatory response is not merely a concoction of non-specific responses and phagocytosis. Rather it is the main orchestrator of the subsequent adaptive responses and is able to sense pathogen associated molecular patterns (PAMPs) with a specificity which was previously unsuspected. In inflammatory conditions, including atherosclerosis, the immune inflammatory apparatus is chronically activated, either due to the persistence of pro-inflammatory stimuli or due to the failure of regulatory mechanisms that should facilitate resolution. Significant progress has been made in the field linking innate immune sensors to the recognition of cholesterol [21] and modified lipoproteins [22-24]. Thus diverse innate immune signalling pathways have been seen to cooperate to induce and maintain inflammation upon exposure to exogenous and, importantly, endogenous molecular patterns [21,25].

The most abundant cell types within the atherosclerotic plaque are innate immune cells, such as monocyte-macrophages, dendritic cells (DCs) and mast cells. Monocytes-macrophages came to the forefront of research owing to new awareness that they may represent a more heterogeneous and phenotypically plastic population than previously anticipated. In this review we focus on the role of macrophage activation and phenotypic polarisation in lesion formation and vulnerability.

### Macrophage heterogeneity in atherosclerosis

Macrophages are a heterogeneous population of cells that adapt in response to a variety of micro-environmental signals; their phenotype is very much a function of environmental cues [26,27]. In a nomenclature mirroring Th1 and Th2 polarisation, macrophages are usually defined as M1 or M2 [28]. Classically activated (M1) macrophages were the first to be defined [29,30] as pro-inflammatory. Alternatively activated (M2) macrophages have been originally characterised in the context of Th2-type immune responses [29]. Subsets of M2-like macrophages have been later found to contribute to wound healing and regulation of inflammatory processes [31]. Characteristic cytokine and chemokine signatures pertaining to human monocyte-to-macrophage differentiation and M1/M2 macrophage polarisation (Table 1) have been described [28,32].

**Table 1:** Cytokine and chemokine genes, and those of receptors (in italics), known to be differentially transcribed in human M1 and M2 macrophage *in vitro *polarisation (Adapted from [28] and [27]).

M1 > M2	M2 > M1
CXCL11	Insulin-like growth factor 1
CCL19	CCL23
CXCL10	CCL18
Tumour necrosis factor ligand superfamily, member 2	CCL13
CCL15	Bone morphogenic protein 2
Interleukin 12B	Hepatocyte growth factor
Interleukin 15	Fibroblast growth factor 13
Tumour necrosis factor ligand superfamily, member 10	CXCL1
Interleukin 6	*Transforming growth factor β receptor II*
CCL20	*CXCR4*
Visfatin	*Mannose receptor C type 1 (CD206)*
Endothelial cell growth factor	
CCL1	
CCL17	
CCL22	
CCL13	
Transforming growth factor β2	
*CCR7*	
*Interleukin 2 receptor α chain*	
*Interleukin 15 receptor α chain*	
*Interleukin 7 receptor*	

CCL2 was upregulated in M-CSF differentiated macrophages in one study [27], whilst relatively increased by GM-CSF in another [28].

**Figure 1:**
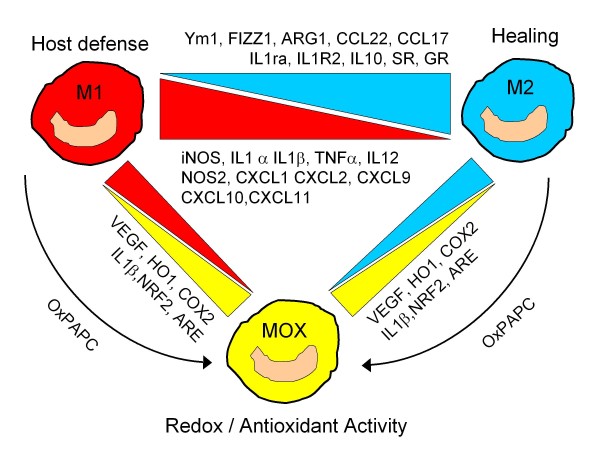
**Macrophages have classically been described as M1 and M2**. These two phenotypes differ substantially with respect to the expression of macrophage associated genes. More recently, Kadl *et al *have described a new subset termed MOX macrophages [36]. These are induced by an environment rich in structurally defined oxidation products such as oxidised 1-palmitoyl-2-arachidonoyl-sn-3-phosphorylcholine (oxPAPC) and can be induced from an M1 or M2 phenotype. ARE, antioxidant responsive elements; ARG1, arginase 1; CCL, chemokine ligand; COX2, cyclo-oxygenase 2; CXCL, chemokine CXC motif ligand; FIZZ1, found in inflammatory zone 1; GR, galactose receptor; HO1, heme-oxygenase 1; IL, interleukin; IL1ra; interleukin 1 receptor antagonist; ILR2, interleukin 1 receptor type II, decoy receptor; iNOS, inducible nitric oxide synthase; NRF2, nuclear factor erythroid 2-like 2; SR, scavenger receptor; Ym1, chitinase 3-like 3 lectin.

Macrophage phenotypic polarisation may have a role in the fate of an atherosclerotic plaque. The plaque is an environment with a strong skew towards Th1 lymphocytic responses, resulting in high levels of IFNγ [33,34] which could in theory privilege M1-type macrophage polarisation. However, studies thus far have demonstrated macrophage heterogeneity within atherosclerosis, supporting that both M1 and M2 macrophages are present in human and murine atherosclerotic lesions. In an ApoE^-/- ^murine model of atherosclerosis, early lesions were seen to be infiltrated by M2 (arginase I^+^) macrophages [35]. As lesions progressed a phenotypic switch was observed, with an eventual predominance of M1 (arginase II^+^) macrophages. Upon exposure to the oxidised phospholipid 1-palmitoyl-2-arachidonoyl-sn-3-phosphorylcholine (oxPAPC), murine macrophages adopted a previously undescribed phenotype (Figure 1) [36]. A reduction in the expression of genes characteristic of both M1 and M2, coupled with an up-regulation of a unique redox gene signature that includes haemoxygenase 1, was observed. This population, termed Mox macrophages, are nuclear factor erythroid 2-like 2 (Nrf2)-dependent and have been shown to comprise approximately 30% of all macrophages in advanced atherosclerotic lesions of LDLR^-/- ^mice [36]. A variety of subtypes have been described which are considered to fall under the umbrella of alternatively activated M2 macrophages (reviewed in [31,37]). An example of this occurs with administration of IL33 (which is functionally atheroprotective [38]) to genetically obese diabetic (*ob/ob*) mice, resulting in increased production of Th2 cytokines and polarisation of adipose tissue macrophages to a CD206^+ ^M2 phenotype [39].

In human lesions different macrophage phenotypes exist, and do so in different plaque locations. M2 (CD68^+ ^CD206^+^) macrophages were seen to reside in areas more stable zones of the plaque distant from the lipid core, with their M1 (CD68^+ ^CCL2^+^) counterparts displaying a distinct tissue localisation pattern [40]. Subsequent work has confirmed this, finding CD68^+ ^CD206^+ ^cells far from the lipid core [41]. CD68^+ ^CD206^+ ^macrophages were also seen to contain smaller lipid droplets compared to CD68^+ ^CD206^- ^[41]. A subset of M2 macrophages has recently been detected in association with intraplaque haemorrhage in coronary atheromata [42]. These macrophages express high levels of CD163 (a scavenger receptor that binds to haemoglobin-haptoglobin (HbHp) complexes). They also express low levels of MHC Class II and display low release of the reactive oxidative species hydrogen peroxide. Expression of CD163 by peripheral blood monocytes was not shown to be different between the CD14^+ ^CD16^+ ^and CD14^++ ^CD16^- ^subsets. However, when monocytes were differentiated into macrophages in the presence of HbHp complexes for 8 days, they matured into a CD163^high ^HLA-DR^low ^phenotype similar to the haemorrhage-associated macrophages within coronary plaques [42]. Differentiation into this macrophage subtype was dependent on the expression of CD163 and IL10 during in vitro blockade experiments. Interestingly, this polarisation was prevented by the incubation with specific inhibitors of endolysosomal acidification, such as chloroquine which is known to interfere with endosomal TLR signalling [42].

Lesion development and stability are not only determined by the influx and differentiation of inflammatory cell subsets, but also their ability to act on vascular extracellular matrix. Importantly, the macrophage subtypes display a differential expression of matrix metalloproteinase (MMP) and tissue inhibitor of metalloproteinase (TIMP) [43]. In particular, a subset of lesional foam cell macrophages characterised by a high expression of MMP14 (membrane type 1 MMP) and a low expression of TIMP3 were highly invasive and catabolic [44]. Moreover, such expression pattern of MMP14 and TIMP 3 was associated with markers of M1 polarisation [44], whilst expression of MMP12 was associated with an M2-typical down-regulation of arginase I [45]. Thus MMP expression by macrophage subsets is also heterogeneous, further highlighting the different functionalities of these cells.

The heterogeneity of macrophage phenotypes in the various studies is an important feature of our current view of atherosclerosis. Studies assessing multiple markers in human and murine lesions are needed to map such degree of heterogeneity. How is such heterogeneity generated? It is likely to be the result of recruitment of different monocytes subsets, or stimuli provided by the plaque microenvironment. Gordon and Martinez have proposed a four-stage paradigm of macrophage activation, where differentiation through exposure to growth factors is the first stage [46]. This stage is followed by priming (through cytokines, particularly IFNγ and IL4), activation (by TLR or similar), and finally resolution and repair (mediated by IL10, transforming growth factor (TGF)-β, nucleotides, glucocorticoids or lipotoxins) [46]. This review will explore the potential mechanisms leading to macrophage activation and polarisation in atherosclerosis.

## Recruitment of monocyte subsets to atherosclerotic plaques

In both mice and humans, monocytes comprise 5 to 10% of peripheral blood leukocytes [25]. Two major circulating monocyte subsets have been described in humans and mice alike, the distinction made on the basis of size, granularity, and the differential expression of chemokine receptors and adhesion molecules [47]. The two mouse monocyte sub-populations are represented approximately equally in murine blood; they are distinguished based upon their expression of CCR2, CX_3_CR1 and Ly6C [48]) [49]. CCR2^+ ^CX_3_CR1^low ^Ly6C^+ ^monocytes are termed 'inflammatory' monocytes, and CCR2^- ^CX_3_CR1^high ^Ly6C^- ^are referred to as 'resident' monocytes [31,47,50].

Similarly to mouse monocytes, human monocytes can be separated into two groups based upon cell surface CD14 - a toll-like receptor (TLR) co-receptor sensing exogenous molecular patterns such as lipopolysaccharide (LPS) - and CD16 - a member of the family of Fc (Fragment, crystallisable) receptors FcγRIII. In humans, about 90% of monocytes are CD14^++ ^CD16^- ^and termed 'classical' monocytes [50,51]. CD14^+ ^CD16^+ ^monocytes, which constitute the remaining minority, are referred to as 'non-classical' [52-55] (Table 2).

**Table 2:** A comparison of human and murine monocyte subsets, highlighting differences in surface receptor phenotypes.

	Human	Mouse
**Classical/Inflammatory**	CD14^++ ^CD16^- ^[195,196] (>90%)	Ly6C^+ ^CCR2^+ ^CD62L^+ ^CX_3_CR1^low ^[47,59] (~50%)
**Non-Classical/Resident**	CD14^+ ^CD16^+ ^[195,196] (<10%)	Ly6C^- ^CCR2^- ^CD62L^- ^CX_3_CR1^high ^[47,59] (~50%)

The approximate abundance in peripheral blood is shown in brackets, however this may not reflect the proportions in other sites such as the spleen.

To date, monocyte phenotype data has centred largely on the murine system [29]. Similarities between mice and humans may be accounted for, at least in part, by the expression of surface receptors. For instance, chemokine receptors CCR1 and CCR2 are highly expressed on both CD16^- ^human and Ly6C^+ ^murine monocytes, and CX_3_CR1 is increased on CD16^+ ^human and Ly6C^- ^mouse monocytes [47,56,57] (reviewed in [58]). More than 130 of these gene expression differences were conserved between mouse and human monocyte subsets, with many of these differences also confirmed at the protein level [59]. A notable difference among these was the high expression of peroxisome proliferator-activated receptor γ (PPARγ, discussed in greater detail below) in Ly6C^- ^mouse monocytes, but not the proposed CD16^+ ^counterpart [59]. As such, the differences between mouse and human monocyte subsets may be greater than had been expected and may be difficult to reconcile.

Two groups independently reported in 2007 that the Ly6C^+ ^inflammatory monocyte subset increases its representation dramatically in the peripheral blood of the hypercholesterolemic apolipoprotein E (ApoE) deficient mouse on a high-fat diet [56,60]. Conversely, hypercholesterolemia did not affect Ly6C^- ^monocytes and also discouraged the conversion of Ly6C^+ ^into Ly6C^- ^monocytes. Other mechanisms proposed for this increase in Ly6C^+ ^monocytes during hypercholesterolemia include increased proliferation and reduced apoptosis [61]. Ly6C^+ ^monocytes are recruited to activated endothelium and are thought to represent the majority of infiltrating macrophages within atherosclerotic plaques [60]. Conversely, Ly6C^- ^enter the atherosclerotic plaque in lower numbers and preferentially express CD11c upon entry [56]. This differential recruitment based upon Ly6C expression may condition the macrophage phenotype within the plaque, with reports that Ly6C^+ ^monocytes differentiate into cells that resemble M1 macrophages and that cells derived from Ly6C^- ^monocytes exhibit M2 characteristics [62-65].

Chemokine receptors are necessary for monocytes to traverse the endothelium [56,66] (reviewed in [16]). CX_3_CR1^-/- ^(fractalkine receptor) [67,68], CX_3_CL1^-/- ^(fractalkine) [69] and CCR2^-/- ^[70,71] mice (in the context of low density lipoprotein receptor (LDLR) or ApoE deficiency) exhibited a reduction in - but not elimination of - atherosclerosis. Furthermore, deficiency of CCR5 (the receptor for CCL5, a chemokine also known as RANTES) in ApoE^-/- ^mice does not appear to be protective in the early stages of atherosclerosis [72]. Subsequently, in a wire injury study also using the ApoE^-/- ^mouse model, the authors found a significant reduction in the area neo-intima formation with concurrent CCR5 deficiency, but not with concurrent absence of the alternative CCL5 receptor CCR1 [73]. More recently, a multiple knockout model has reaffirmed the thinking that CCL2 (MCP1), CCR5 and CX_3_CR1 play independent and additive roles in atherogenesis [74]. Combined inhibition of CCL2, CCR5 and CX_3_CR1 in ApoE^-/- ^mice results in a 90% reduction in atherosclerosis, which is related to progressive monocytopaenia [66,74]. However, chemokine receptor utilisation during recruitment to atherosclerotic plaques differentiates Ly6C^+ ^and Ly6C^- ^monocytes. Ly6C^+ ^monocytes are recruited to mouse atherosclerosis via CCR2, CCR5 and CX_3_CR1 [61]. Conversely, Ly6C^- ^monocytes are recruited less frequently and through CCR5.

In human atherosclerosis, patients with coronary artery disease have increased numbers of circulating CD14^+ ^CD16^+ ^monocytes compared to controls [75]. Furthermore, these patients have raised levels of serum TNFα [76]. There is, however, data to the contrary with the finding that inflammatory genes and surface markers were down-regulated in monocytes of patients with coronary atherosclerosis [77]. Of relevance, CD14^+ ^CD16^+ ^monocytes have also been shown to exhibit pro-inflammatory and pro-atherosclerotic activity in a population of elderly human subjects. These activated monocytes exhibited increased interaction with endothelium and had higher expression of chemokine receptors [78]. Other studies have suggested that the bone marrow is the source of these monocytes [79,80].

### Macrophage differentiation in atherosclerosis

Early work relating to the effect of the colony stimulating factors (CSFs) on macrophage phenotype was undertaken by Hamilton and colleagues [81,82]. A variety of groups have generated data using monocytes differentiated *in vitro*, via exposure to either M-CSF or GM-CSF [82,83]. *In vitro *differentiation with M-CSF results in a macrophage phenotype close to that of M2 [28]. GM-CSF plays a role in the induction of a pro-inflammatory macrophage phenotype that resembles M1 polarisation, proficiently producing inflammatory cytokines such as TNFα and IL6, and being involved in tissue destruction [28].

In further murine studies, both M-CSF and GM-CSF have been shown to be important in plaque development. Smith *et al *studied ApoE^-/- ^mice crossbred with the osteopetrotic mutation of the M-CSF gene. These mice were fed a low-fat chow diet with the double mutants exhibiting significantly smaller proximal aortic lesions, at an earlier stage of progression and with fewer macrophages as compared with their control ApoE^-/- ^littermates [84]. The production of GM-CSF from smooth muscle cells leads to the activation of monocytes during atherogenesis [85]. In another study using the hypercholesterolaemic ApoE^-/- ^mouse, animals on a high-fat diet were injected with doses of 10 μg/kg GM-CSF or G-CSF daily for 5 days on alternating weeks for a total of 20 doses during an 8 week period, finding that both G-CSF and GM-CSF treatment resulted in increased atherosclerotic lesion extent [86]. LDLR-null mice have been employed in a study which combined 5-bromo-2'-deoxyuridine pulse labelling with *en face *immunoconfocal microscopy to demonstrate that systemic injection of GM-CSF markedly increased intimal cell proliferation, whilst functional GM-CSF blockade inhibited proliferation [87].

In a key study, Waldo and colleagues examined human macrophages differentiated *in vitro *for 7 days with either M-CSF or GM-CSF [27]. They characterised gene expression, surface phenotype, cytokine production and lipid handling in these two macrophage groups. With regards to gene expression, they demonstrated differential expression of genes of inflammation (Table 1) and cholesterol homeostasis between the two groups, including that GM-CSF macrophages exhibited a ten-fold increased gene expression of PPARγ. M-CSF differentiated macrophages spontaneously accumulated cholesterol when incubated with unmodified low density lipoprotein (LDL), whilst GM-CSF differentiated macrophages took up similar levels only when exposed to protein kinase C. Macrophages differentiated with M-CSF were shown by immunofluoresence to express CD14 (CD68^+ ^CD14^+^), whilst GM-CSF differentiated macrophages were CD68^+ ^CD14^-^. Interestingly, human coronary plaque samples were shown to contain predominantly CD68^+ ^CD14^+ ^[27].

### Priming of macrophages in the atherosclerotic plaque

Macrophages are M1-primed by exposure to interferon (IFN)-γ [37]. The key role of IFNγ [88] has been confirmed in experimental atherosclerosis whereby ApoE^-/- ^IFNγ receptor^-/- ^mice displayed a substantial reduction in lesion size compared to ApoE^-/- ^[89]. This reduction was manifest alongside a reduced level of macrophages and T lymphocytes within the lesions. Furthermore, murine cardiac allografts sited in IFNγ^-/- ^recipients had reduced transplant atherosclerosis [90].

Alternative M2 polarisation has originally been described as the result of exposure to interleukin (IL4) [28,40,58,91]. M2 macrophages have a notable role in catabasis, the process inflammation resolution which when fails results in progression of atherosclerosis [92].

Wound healing macrophages, concerned primarily with tissue repair, are similar to the alternatively activated (M2) macrophages which have been described above. Wound healing macrophages establish their phenotype upon exposure to IL4 and/or IL13 from Th2 cells and granulocytes. IL4 is an early innate signal released during tissue injury, stimulating macrophage arginase to convert arginine to ornithine which is a step in extra-cellular matrix collagen production [93]. This ornithine is a precursor for polyamines which have an effect on cytokine production, affording wound healing macrophages regulatory capabilities [94].

Regulatory macrophages, with anti-inflammatory activity, are most reliably defined and identified through IL10 levels or IL10/IL12 ratio (as they also downregulate IL12 [95]). These develop in response to a large number of stimuli, including IL10 produced by regulatory T cells, TGFβ [96], and glucocorticoids. The latter attenuate macrophage-mediated inflammation through inhibition of pro-inflammatory cytokine gene transcription [97], nonetheless capacity for phagocytosis does not appear to be impaired by glucocorticoids [98]. Unlike wound-healing macrophages, regulatory macrophages do not contribute to the production of extracellular matrix.

### Macrophage activation pathways in atherosclerosis

Following the priming stage, activation of macrophages is reliant upon ligation of pattern recognition receptors (PRR) [29,99], namely nucleotide-binding oligomerisation domain (NOD)-like receptors (NLRs) and TLRs.

#### Toll-like receptor signalling

TLRs are the most well-characterised PRRs, of which at least ten have been identified in humans [100]. TLRs may be found on the cell surface, as in the case of TLRs 1, 2, 4, 5 and 6, or reside intracellularly [101,102]. TLRs are key activators of monocytes and macrophages.

**Figure 2:**
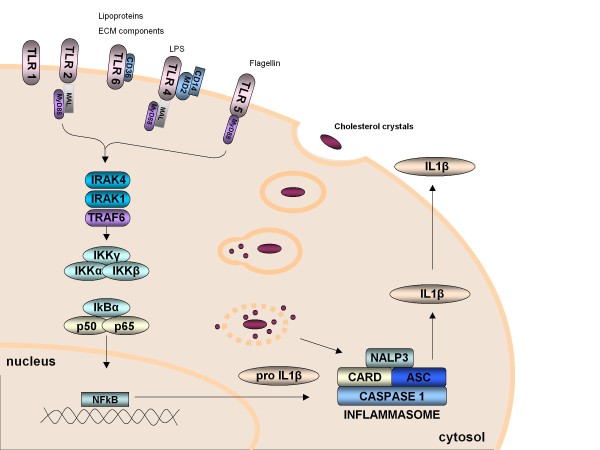
**The interaction between innate signalling, through TLRs, and inflammasome signalling in the transcription and translation of the pro-inflammatory cytokine IL1**. Oxidised LDL is a ligand for TLR, resulting in IL1 RNA transcription. Inflammasomes (which may be activated by cholesterol crystals [21]) initiate intracellular pathways which result in the post-translational modification and, ultimately the secretion of IL1 protein. Therefore, a connection between TLR and inflammasome pathways in the innate inflammatory process in atherosclerosis is alluded to. ASC, apoptosis-associated speck-like protein containing a CARD; CARD, caspase recruitment domain; CD, cluster of differentiation; ECM, extra-cellular matrix; IκB, nuclear factor of kappa light polypeptide gene enhancer in B-cells inhibitor; IL, interleukin; IRAK, interleukin 1 receptor-associated kinase; LPS, lipopolysaccharide; MAL, MyD88 adaptor-like; MyD88, myeloid differentiation primary response gene 88; NALP3, nucleotide-binding oligomerization domain-like receptor P3; NFκB, nuclear factor kappa B; PAMPs, pathogen-associated molecular patterns; TLR, toll-like receptor, TRAF, tumour necrosis factor receptor associated factor.

Upon exposure to ligand, TLRs couple to signalling adaptors to induces two major downstream signalling pathways: the nuclear factor kappa B (NFκB) (Figure 2) and the interferon response factor (IRF) pathways. MyD88 is a universal adapter protein that carries signalling through all TLRs, except TLR3, leading to the activation of NFκB. MyD88-dependent signalling relies on recruitment of Mal (MyD88-adaptor like), which leads to the recruitment of the IL1 receptor-associated kinase (IRAK). Phosphorylation of IRAK signals to tumour-necrosis-factor-receptor-associated factor 6 (TRAF6). The subsequent nuclear translocation of NFκB and translation of inflammatory cytokines is driven by phosphorylation of the IκB kinase (IKK) complex upon activation of TRAF6. MyD88-independent signalling is via TRAM (TRIF-related adaptor molecule) and TRIF (TIR-domain-containing adaptor protein inducing IFNβ), and can activate both NFκB and IRF, inducing interferon synthesis. The importance of IL1/TLR signalling in atherosclerosis has been further highlighted by work implicating IRAK4 kinase in modified LDL-medicated experimental atherosclerosis [103].

The most characterised recognition system is the one sensing LPS. Serum LPS-binding protein (LBP) transfers LPS to CD14, which delivers it to the co-receptor MD2 [104,105]. The availability of all members of the complex dictates the sensitivity of recognition of endotoxin at extremely low concentrations. Cells that do not express CD14, such as endothelial cells, are relatively unresponsive compared to CD14^+ ^monocytes [104,105]. CD14 acts as a co-receptor (along with TLR4 and MD2) for the detection of bacterial LPS. CD14, however, can only bind LPS in the presence of LBP. TLR2 may also be activated via scavenger co-receptors, including CD36 [106].

#### Toll-like receptor agonists

Initially, ligands binding to PRRs such as TLRs on/in innate immune cells were believed to be of a pathogenic aetiology; molecules or small molecular motifs derived from, conserved within or associated with groups of microorganisms (such as bacterial LPS). These have been nominated pathogen associated molecular patterns (PAMPs). More recently, such ligands have been classified as danger associated molecular patterns (DAMPs) encompassing a wider definition which embodies the existence of endogenous danger signals. The concept that oxidation reactions involving lipids, proteins and DNA produce non-microbial 'oxidation-specific epitopes' has emerged [107]. Of particular interest is that host-derived oxidation-specific epitopes represent endogenous DAMPs, are recognised by PRRs and are capable of driving the inflammation seen in atherosclerosis [107].

DAMPs that may bind TLRs are numerous, some of which have been proposed as endogenous culprits in atherosclerosis. Examples of endogenous ligands to TLR2 include necrotic cell products [108], apolipoprotein CIII [109], serum amyloid A [110], versican [111]. Furthermore, oxidised phospholipids, saturated fatty acids, and lipoprotein A have been shown to trigger macrophage apoptosis, under conditions of thapsigargin-induced endoplasmic reticulum stress, via a mechanism requiring both CD36 and TLR2 [112].

Hyaluronan fragment [113], biglycan [114], oxLDL [115,116] and heat shock proteins [117] have been shown to act through both TLR2 and TLR4. Long surfactant protein A [118], tenascin C [119], fibrinogen [120], fibronectin EDA [121], heparan sulphate [122], β-defensin 2 [123], amyloid β peptide [24] and minimally modified LDL (mmLDL) [23] act via TLR4 alone. TLR3 detects mRNA [124,125], whilst TLR7 and TLR9 detect nucleic acid-containing immune complexes [126,127]. TLRs 5, 6 and 8 are yet to have endogenous ligands allocated to them [25].

Although both mmLDL and oxLDL are seen as ligands to TLR4, the pathways by which recognition occurs differ. The recognition of mmLDL is similar to that of LPS and involves CD14 and MD2 [22], whilst oxLDL initiates inflammatory responses through a TLR4/TLR6 heterodimer in association with CD36 but independently of CD14 [128]. A lipidic component of LDL, namely oxPAPC, has been shown as capable of inducing IL8 transcription via TLR4 in a manner which is independent of both CD14 and CD36 [129]. Further work, however, has seen oxPAPC inhibiting TLR4-dependent IL8 induction, along with inhibition of E-selectin and CCL2, whilst IL1β and TNFα signalling remained unhindered [130]. Downstream of TLR4/MD2/CD14, intracellular signalling in response to mmLDL stimulation has been investigated and, in addition to the canonical MyD88 pathway, an alternative pathway via sequential activation of spleen tyrosine kinase (Syk), phospholipase Cγ1, protein kinase C, and NADPH oxidase 2 (gp91phox/Nox2) has been proposed in the stimulation of pro-inflammatory cytokine production and the effects thereof [131].

#### Toll-like receptor expression in atherosclerosis

TLRs are differentially expressed by the various cell types in atherosclerosis, with TLR2 and TLR4 found on monocytes, macrophages, foam cells and myeloid DCs, as well as smooth muscle cells and B lymphocytes (reviewed by [25]). Human and mouse atherosclerosis is characterised by an increased expression of TLR1, TLR2 and TLR4 (and to some extent TLR5), mainly by macrophages and endothelial cells [116,132]. In mouse atherosclerosis, TLR4 expression is exclusively by macrophages [116]. There has been shown to be co-localisation of p65 (an NFκB family member) with both TLR2 and TLR4 in macrophages in atherosclerosis [132].

The differential expression of the various TLRs by monocyte subsets and macrophage subtypes remains largely unknown at present, however there is some data to support the relative transcription of TLR5 being higher in M2 polarised human macrophages as compared with M1 [28]. The circulating monocytes of ApoE^-/- ^mice with advanced atherosclerosis have increased TLR2 and TLR4 expression [133]. This is also the case for monocytes from patients with arterial disease when comparison is made with controls subjects [134-137]. Interestingly, enhanced TLR signalling is restricted to patients with acute coronary syndromes [138-140].

#### Role of Toll-like receptors in atherosclerosis

When recognising ligands, the majority of TLRs associate the signalling adaptor MyD88 to initiate an intracellular signalling cascade. More specifically, removing the MyD88 pathway led to a reduction in aortic atherosclerosis (by approximately 60%) and a decrease in macrophage recruitment to the artery wall (by approximately 75%), associated with reduced chemokine levels [141,142]. In a functional human atherosclerosis study, a significant reduction of pro-inflammatory cytokines and MMPs was found after MyD88 inhibition [143].

The role of TLR2 and TLR4 has been extensively studied in models of atherosclerosis. The first indication of a role for TLR4 in atherosclerosis came from the finding that C3H/HeJ mice - that hold a missense mutation of TLR4's cytoplasmic component - are resistant to atherosclerosis [144,145]. In accordance, specific deletion of TLR4 in ApoE^-/- ^mice resulted in a 24% reduction in whole aortic atherosclerotic lesion area and significantly attenuated macrophage infiltration within these lesions [141]. TLR2 deletion in LDLR^-/- ^mice limits lesion area by between a third and two-thirds [141,146-148], reducing intra-lesion inflammation as evidenced by a reduction in total infiltrating macrophage numbers [147,148], and attenuates macrophage to smooth muscle cell ratio and extent of apoptosis [147].

Both TLR2 and TLR4 are known to be important in post-vascular injury neo-intimal lesion formation [149,150]. In a hypercholesterolaemic rabbit model of atherosclerosis, carotid artery liposomal transfection of TLR2 and TLR4 cDNA revealed that upregulation of either TLR *alone *did not significantly affect carotid atherosclerosis. Interestingly, transfection of both TLR2 and TLR4 together resulted in a synergistic acceleration of atherosclerosis [151]. Recently, LDLR^-/- ^mice transplanted with TLR2^-/- ^TLR4^-/- ^bone marrow displayed a reduction in both macrophage apoptosis and atherosclerotic plaque necrosis as compared with LDLR^-/- ^mice transplanted with wild-type bone marrow, supporting an additive effect of TLR2 and TLR4 in murine atherosclerosis [112].

A different picture came from bone marrow chimera studies. Bone marrow transplantation from TLR2^-/- ^to LDLR^-/- ^mice was unable to prevent diet-induced atherosclerotic lesions [146]. Bone marrow transfer from C3H/HeJ to ApoE knockouts did not alter atherosclerosis susceptibility [152]. Synthetic TLR2 ligand administered dramatically increases atherosclerosis in LDLR^-/- ^mice, with TLR2 deficient bone marrow transfer into this model preventing TLR2 ligand-induced atheroma [146]. Such studies raise the question of whether TLR2 signalling in myeloid cells is relevant in atherosclerosis, as compared with TLR2 expression by cells resident in the arterial wall. Importantly, it supports the role of endogenous TLR2 ligand action on myeloid cells in atherosclerosis, with exogenous agonists activating TLR2 on cells of a non-myeloid lineage.

What are the mechanisms through which TLR exert proatherogenic actions? Importantly, TLR2, TLR4 and TLR9 ligands promote lipid uptake by macrophages and, hence, foam cell formation [111,153-155]. Differentiated macrophages exhibit macropinocytosis (fluid phase uptake of lipids) which is dependent upon TLR4 [156]. However, the effect of TLR signalling are not limited to foam cell formation but have a direct effect on inflammation and matrix degradation.

Functional studies on human carotid endarterectomy specimens have shown sustained TLR2 activation in cells isolated from human atheromata [143]. TLR2 and MyD88 play a key role in NFκB activation, and in the production of inflammatory mediators CCL2, IL6, IL8, MMPs 1, 2, 3 and 9 [143]. Conversely TLR4, and its downstream signalling adaptor TRAM, were shown not to be rate-limiting for cytokine production in this context. This adds weight to the role of some (but not all) TLRs in plaque vulnerability.

Furthermore, and as alluded to above, TLR ligation may influence atherosclerosis through alterations in MMP and TIMP expression. The effect of LPS on human blood monocytes has been investigated and MMP3 is upregulated [157], whilst MMPs 1, 2, 7, 10 and 14 and TIMPs 1, 2 and 3 are not upregulated by LPS [157,158]. Controversially, two separate studies have found upregulation [159] and no upregulation [157] of MMP9 in human blood monocytes stimulated with LPS. In human macrophages (from various sites) meanwhile, MMPs 2, 3, 8, 9 and 14, and TIMP1 have all been upregulated by LPS [158,160-163].

Using both human and murine models of atherosclerosis, we have investigated the consequence of endosomal TLRs in atherosclerosis and arterial injury. Deficiency of TLR3 accelerates the onset of atherosclerosis in ApoE^-/- ^mice. Moreover, genetic deletion of TLR3 dramatically enhanced the development of elastic lamina breakages after collar-induced injury. The systemic (intraperitoneal) administration of double-stranded RNA (dsRNA) - a TLR3 agonist - decreased neointima formation upon arterial injury. Genetic deletion of TLR3 was associated with the presence of large interruptions of the elastic lamina after the placement of a perivascular collar for arterial injury development. Finally, lesion development in both human and mice was associated in an increase of expression of TLR3 and TLR3-associated responses, in particular in smooth muscle cells pointing to this cell type as the carrier of the protective effect. This data shows for the first time that while extracellular TLRs may be detrimental to atherosclerosis, intracellular TLRs may offer protection against hypercholesterolemia and injury-induced lesions. The mechanism of TLR3-induced protection is currently unknown. IFNβ production - that is a consequence of TLR3 dependent signalling - has been associated with a reduction in inflammasome activation and IL1 signalling, as well as with induction of IL10 [164]. However, it is uncertain whether the vasculoprotective effect of TLR3 may be mediated via IFNβ. Although IFNβ has been shown to be effective in an arterial injury model [165], a more recent report showed a potential deleterious role in atherosclerosis induced by hyperlipidemia [166]. It is also uncertain whether synthetic dsRNA is safe as therapeutic tool, as its administration elicits both pro-inflammatory and anti-inflammatory mediators [124]. Moreover, a recent study showed that dsRNA intravenous administration at high doses may lead to endothelial cell apoptosis and increased vascular lesion formation [167]. Further studies are needed to elucidate the mechanisms of vasculoprotection elicited by TLR3. TLR3 activation has been shown to elicit the production in the vasculature of IL10 [124] and of the B7 family members programmed cell death ligands PDL1 and PDL2, which are known to contribute to vascular protection [168,169].

It is also unknown what endogenous agonists of TLR3 may be involved in protection, as the genetic removal of TLR3 accelerates atherosclerosis and elastic lamina damage. Interestingly, stathmin, a protein that participates in microtubule assembly and is upregulated in brain injury, has been described as a candidate TLR3 agonist and has been linked to the induction of a neuroprotective gene profile [170].

#### NOD-like receptors and inflammasomes and atherogenesis

NLRs are PRRs that sense intra-cellular microbial and non-microbial signals, in a similar fashion to the extra-cellular detection of these entities by most TLRs. NLRs have the capacity to form large cytoplasmic complexes known as "inflammasomes" (reviewed in [171]) through the assembly of NLRs, caspase and apoptosis-associated speck-like protein containing a caspase recruitment domain (ASC). ASC acts to link the NLR and caspase, the latter of which are usually caspase 1 and 11 [172]. The inflammasome acts as a scaffold for the activation of caspase 1 as its central effector molecule [173]. Upon activation, inflammasome caspase 1 proteolytically activates pro-inflammatory cytokines, notably the conversion of pro-IL1β and pro-IL18 to IL1β and IL18, respectively.

It is largely agreed that inflammasome activation resulting in active IL1β release requires two separate signals [174]. A priming signal may be triggered by TLR activation, with resultant NFκB production leading to pro-IL1β synthesis, as well as inflammasome components such as caspase 11 [173]. Recognition of peptidoglycan by NOD1 and NOD2 can also trigger activation of NFκB signal transduction through Rip2 kinase [100]. The second signal, which activates the caspase 1 of a complete inflammasome, allowing the conversion of available pro-IL1β to IL1β includes activation by ATP of the P2X_7 _purinergic receptor with potassium efflux. The second signal may also be achieved by PAMPs such as bacterial toxins and viral DNA, or other DAMPs including oxidative stress, large particles and ultraviolet light [171].

Inflammasomes have been described in a number of inflammatory conditions [171] and evidence for their role in atherosclerosis is emerging. The NLRP3 inflammasome is currently the most characterised inflammasome (Figure 2). Recent work has shown that cholesterol crystals activate the NLRP3 inflammasome, which in turn results in cleavage and secretion of IL1 family cytokines [21]. Furthermore, LDLR-deficient mice transplanted with NLRP3-deficient bone marrow and fed a high-cholesterol diet had markedly decreased early atherosclerosis and inflammasome-dependent IL18 levels [21]. LDLR^-/- ^mice bone-marrow transplanted with ASC-deficient or IL-1α/β-deficient bone marrow and fed on a high-cholesterol diet had consistent and marked reductions in both atherosclerosis and IL18 production [21]. Furthermore, ASC deficiency also attenuates neointimal formation after vascular injury via reduced expression of IL1β and IL18, with ASC^-/- ^bone marrow chimeras also exhibiting significantly reduced neointimal formation [175]. These findings taken together suggest that crystalline cholesterol acts as an endogenous danger signal, its deposition in arteries being an early cause rather than a late consequence of inflammation.

Both IL1 and IL18 signal through MyD88, and their absence in experimental mouse atherosclerosis also has the effect of limiting atherosclerosis development [176,177]. Devlin *et al *showed that IL1ra knockout mice on a cholesterol/chocolate diet, exhibited a 3-fold decrease in non-high-density lipoprotein (HDL) cholesterol and a trend toward increased foam cell lesion area compared to controls [178]. Complementing this experiment they showed, conversely, that increased IL1ra expression (using an IL1ra transgenic/LDLR^-/- ^mouse on a cholesterol-saturated fat diet) resulted in a 40% increase in non-HDL cholesterol levels. Thus concluding that under certain conditions, chronic IL1ra depletion or over-expression could have an important effect on lipid metabolism.

This was also verified in human atherosclerotic arteries [179], although more recently, IL1ra administration has been shown to have lesser effect on inflammatory molecule production when compared to TLR inhibition in the context of human atherosclerosis [143].

### Macrophage deactivation pathways in atherosclerosis

**Figure 3:**
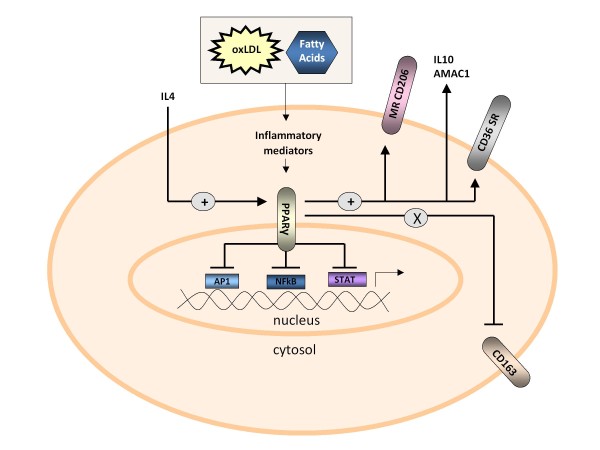
**Peroxisome proliferator-activated receptor γ (PPARγ) is a ligand-activated nuclear receptor with potent anti-inflammatory properties that modulates the immune inflammatory response**. It has been observed in human atherosclerotic lesions and is involved in macrophage cholesterol homeostasis, cellular differentiation, lipid storage, insulin modulation, macrophage lipid homeostasis and anti-inflammatory activities. Molecules such as oxidised low density lipoprotein (oxLDL) or fatty acids may stimulate inflammatory mediators such as 9- and 13- hydroxyoctadecadienoic acid (HODE) generated via the 12,15 lipoxygenase pathway. These are ligands for PPARγ. IL4 is a cytokine that can stimulate PPARγ. PPARγ activation is also associated with the expression of M2 macrophage markers such as the mannose receptor (MR) also known as CD206 [40]. AMAC1, alternative activated macrophage associated CC-chemokine 1; AP1, activator protein 1; CD, cluster of differentiation; IL, interleukin; NFκB, nuclear factor kappa B; SR, scavenger receptor; STAT, signal transducer and activator of transcription.

PPARγ has recently been highlighted as an important determinant of macrophage phenotype and function (Figure 3), which may explain the favourable effect of PPARγ modulation in experimental atherosclerosis [180,181]. PPARγ is a ligand-activated nuclear receptor involved in reverse cholesterol transport and other metabolic cellular activities [46]. Its anti-inflammatory properties occur through negative interference with nuclear factor κB (NFκB), signal transducer and activator of transcription (STAT), and activating protein 1 (AP1) pathways [182]. PPARγ is strongly induced by IL4 [40,183]. PPARγ upregulation may also be stimulated by oxidised LDL, with PPARγ being highly expressed in the foam cells of atherosclerotic lesions, and ligand activation of PPARγ promoting oxidised LDL uptake and foam cell formation [184].

The functional relationship between PPARγ and the wound healing M2-type macrophage phenotype [185,186] has been proposed through the positive correlation between PPARγ expression levels and the M2 markers CD206 [187], CD36 scavenger receptor [184], IL10 [188] and alternative activated macrophage associated CC-chemokine 1 (AMAC1; CCL18) [40]. Primary human monocytes differentiated *in vitro *with IL4 in the presence of PPARγ agonist (termed M2γ macrophages) resulted in increased CD206 and reduced CD163 expression, above and beyond that which was seen with IL4 alone [40] (Figure 3). M2γ culture supernatant exerted a greater anti-inflammatory effect on M1 macrophages as compared with M2 culture supernatant [40]. Subsequent work has shown that M2γ macrophages have down-regulation of the nuclear liver × receptor α with resultant enhanced phagocytosis but reduced cholesterol handling [41]. PPARγ also limits MMP9 through inhibition of NFκB activation [189].

However, in the clinical arena, PPARγ agnonists have been shown to have complex and opposing effects on circulating levels of pro- and anti-inflammatory molecules [190-193]. Furthermore, macrophages have been observed adhering to areas of intimal thickening in PPARγ-dependent manner [194].

## Conclusions

Macrophages have been shown to exert a number of diverse functions in atherosclerosis, including inflammation, lipid metabolism and matrix degradation Recent studies have highlighted significant heterogeneity in macrophage behaviour and activation within atherosclerotic plaque. This heterogeneity is derived both from the heterogeneity of originating monocytes, and the inflammatory and lipidic stimuli available in the plaque. It is known that signalling pathways related to innate immunity are strong determinants for macrophage activation and there is growing evidence that they have a significant effect in plaque development and the complications thereof. Innate immune pathways may be activated by both infectious pathogens and endogenous danger signals. An example of the latter is the recognition by innate immune receptors of a growing number of lipoprotein components that are vital to the development of atherosclerosis. Oxidised LDL is seen to signal through TLR [22-24], cholesterol crystals signalling through NLR [21], and oxPAPC signalling via NRF2 [36]. The convergence of these pathways gives rise to the activation of resident monocyte-macrophages leading to cytokine and chemokine production. Moreover, TLR activation might have a role in biasing macrophage polarisation towards an M1 phenotype, together with Th1 lymphocytes present in the plaque. These exciting new findings highlight a wealth of novel potential therapeutic and diagnostic targets that may be exploited in the future treatment of cardiovascular disease.

## Competing interests

The authors declare that they have no competing interests.

## Authors' contributions

All authors were involved directly in the drafting and critical revision of this review. CM is the senior author with overall responsibility for this work, giving the final approval for publication. All authors have read and approved the final manuscript.
